# Regarding “Columella’s” Plan for Controlling Tuberculosis

**Published:** 1902-05

**Authors:** R. G. Rice


### CORRESPONDENCE.

### REGARDING "COLUMELLA'S" PLAN FOR CONTROLLING TUBERCULOSIS.

EDITOR JOURNAL OF COMPARATIVE MEDICINE, ETC.:

THE application of the plan for controlling tuberculosis proposed by "Columella" in the January number of the JOURNAL presents at once insurmountable obstacles.

Having quite an extensive experience in dealing with tuberculosis in cattle, and being familiar with the sentiment of herd owners, I know that the plan would not be practical.

In the first place, persons could not be found who would be willing to take condemned cattle and establish and maintain a herd of them. Investing time and money in that sort of an enterprise would be far from profitable. There is no profit in tubercular cattle. One or more tubercular cows may produce more than the actual expense of keeping them for a short time, but a badly-infected herd of tubercular cattle is a source of loss to the owner; for while a few cows are bringing in a small profit, there are always members of such a herd very badly emaciated, which produce very little, and soon die or have to be destroyed. Why would anyone wish to put time, money, and care on such a herd? I have known of many herds that were conducted in a manner to make them earn a good profit, but owing to tuberculosis these herds did not bring a dollar of profit to the owners for years.

Could anyone be induced to establish such a herd it would be for a short existence only.

In the second place, there appears an objection that could not be overcome: the public would not buy the products of a herd known to be tubercular. While the public might be educated to the fact that their products were not infectious to the extent of producing tuberculosis in the consumer, yet there would not be a demand for "dead bugs," as the milk and butter could be properly said to contain.

I have never seen a method as efficient as the Pennsylvania plan of handling tuberculosis of cattle, and the only chance for improvement would be to procure larger appropriations, so that the work could be carried on more rapidly.

To establish and maintain competent boards as proposed by "Columella" would involve sufficient expense to go a good ways toward paying the herd owners a fair price for their tubercular cattle and having them destroyed. There is no *real value* to a tubercular cow, and while some owners of herds infected with tuberculosis are loath to believe it, or to admit it, for a while, yet when they come to know more of the real facts about this dreadful disease, they are only too willing to have their herds inspected and the reacting cattle destroyed for a small compensation.

And in the localities where the herd owners are the best informed on the subject I find that they would not have the diseased cattle in their herds, even though there was no recompense received for them from the State.

I do not mean to say the method as proposed by "Columella" for controlling tuberculosis in cattle, if it could be put into operation at all, is not better than no plan at all; but I do mean to say that instead of being an improvement over the work as done in Pennsylvania, it is in every way inferior. For, under the management of the State Live-stock Sanitary Board, Pennsylvania is in localities being entirely freed from tuberculosis where it existed to an alarming extent, and if the work can go on for a few years under as good management as it has had by the present board, the last case of tuberculosis in cattle will be disposed of.

All that is needed is that the public shall become better educated as to the true facts regarding tuberculosis, and not only will it be an easy matter to secure necessary legislation to carry on the work properly, but there will not be a market for milk or butter or meat that comes from cattle that are *not known* to be free from tuberculosis.

R. G. Rice, D.V.S.

